# Diabetic kidney disease screening status and related factors: a cross-sectional study of patients with type 2 diabetes in six provinces in China

**DOI:** 10.1186/s12913-024-10938-9

**Published:** 2024-04-19

**Authors:** Zhang Xia, Xuechun Luo, Yanzhi Wang, Tingling Xu, Jianqun Dong, Wei Jiang, Yingying Jiang

**Affiliations:** 1 Department of Epidemiology and Health Statistics, School of Public Health, Capital Medical University, Beijing, China; 2 National Center for Chronic and Noncommunicable Disease Control and Prevention, Chinese Center for Disease Control and Prevention, Beijing, China

**Keywords:** Diabetic kidney disease, Screening, Behavior, Cross-sectional

## Abstract

**Objective:**

To understand the awareness and practice of diabetic kidney disease (DKD) or nephropathy screening among community-based patients with type 2 diabetes in six provinces and cities in China, and to analyse the related factors affecting screening practices.

**Methods:**

From December 2021 to March 2022, a cross-sectional survey was conducted using a structured questionnaire in 6230 patients with type 2 diabetes aged 18 years and older. The content of the questionnaire includes three parts: the general situation of diabetic patients (gender, age, ethnicity, marriage, education, occupation, etc.), DKD screening practices, and the evaluation of DKD screening services.

**Results:**

89.70% of the patients had their fasting blood glucose measured every six months, 21.12% of the patients had their glycosylated hemoglobin measured every six months, and only 13.11% and 9.34% of the patients had a urine protein-creatinine ratio test and estimated glomerular filtration rate test every 12 months. The proportions of glycosylated hemoglobin, urine protein-creatinine ratio, and estimated glomerular filtration rate were relatively high in young, northern, highly educated, and long-duration type 2 diabetic patients.

**Conclusion:**

The results of this survey found that the proportion of urine protein-creatinine ratio testing, estimated glomerular filtration rate testing, and glycosylated hemoglobin testing in Chinese patients with type 2 diabetes was very low. Patients with type 2 diabetes in rural areas, southern areas, with low education level, and short course of disease have lower detection rates for DKD, and hence lower rates of prevention and treatment.

**Supplementary Information:**

The online version contains supplementary material available at 10.1186/s12913-024-10938-9.

## Introduction

Diabetic Kidney Disease (DKD) is caused by diabetes mellitus, increased blood glucose levels. Approximately 40% of people with diabetes may develop DKD [1–4]. Studies in the United States have shown that DKD has accounted for nearly half of all end-stage renal disease. With the rising prevalence of diabetes in China, diabetes-related chronic kidney disease has surpassed glomerulonephritis-related chronic kidney disease and become the primary cause of chronic kidney disease [5]. The latest study showed that in China the overall standardised estimated prevalence of diabetes increased from 10.9% (95% CI, 10.4–11.5%) in 2013 to 12.4% (95% CI, 11.8–13.0%) in 2018 [6]. In China by 2019, 254 million people or 18.1%of the population were aged 60 years and over. By 2040, 402 million people or 28% of the population are estimated to be aged 60 years and over. Diabetes is a progressive disease, and its risk, along with complications such as DKD, increases over time and with the growing aging population [7, 8].

The presence of kidney disease can significantly increase the risk of cardiovascular disease and death, and exacerbate health care costs. Unfortunately, kidney disease usually has an insidious onset, so patients may already have kidney damage when they are diagnosed with type 2 diabetes. Chinese guidelines recommend that patients with type 1 diabetes mellitus (T1DM) with a course of more than 5 years and newly diagnosed type 2 diabetes mellitus (T2DM) patients should be tested for urinary albumin-to-creatinine ratio (ACR) and estimated glomerular filtration rate (eGFR), to enable early detection of DKD, and should be screened at least once a year thereafter [9].

The management service of patients with diabetes in China mainly relies on the national basic public health service. The main services provided are fasting blood glucose measurement every 12 months for high-risk populations, and free fasting blood glucose detection and face-to-face follow-up for diagnosed type 2 diabetes patients every 3 months [10]. Although guidelines and studies have clearly pointed out the necessity of detection and evaluation related to DKD prevention and treatment [11], the implementation of practical is still unclear. Therefore, this study aimed to investigate and evaluate the practice of DKD prevention and treatment in Chinese patients with type 2 diabetes, and their self-evaluation of testing services, in order to provide scientific evidence for the improvement of DKD screening and management services based on patient needs.

## Method

This study adopted a cross-sectional study design and conducted a questionnaire survey of patients with type 2 diabetes in the community aged 18 and over. The study was conducted from early December 2021 to the end of March 2022.

### Investigation sites

Combining the geographical distribution and the level of economic development, Guangdong, Hubei, Chongqing, Tianjin, Heilongjiang and Gansu were selected as study provinces for this study. One urban site and one township site were randomly selected in each province. Medical and health service institutions (township health centres/community health service centres) conducted patient recruitment and questionnaire surveys. The availability of investigators and the cooperation of primary medical and health service institutions were also considered in the selection of the site.

### Subject of investigation

The subjects of this study were adults with type 2 diabetes registered in the local basic public health service system. The diagnostic criteria used are based on the WHO 1999 standards. Inclusion criteria: diagnosed type 2 diabetes patients; 18 years old and above; able to take care of themselves, without obvious limitation of activities, normal consciousness, and able to accept investigator’s inquiries. Exclusion criteria were (a) patients with CKD but not T2DM, (b) patients with T1DM, special type diabetes, and gestational diabetes, and (c) patients with psychiatric disorders.

### Investigation content

The questionnaire was developed by a team of experts and medical professionals in the fields of epidemiology, health promotion, diabetes diagnosis and treatment, and kidney disease diagnosis and treatment. This collaboration was organized by the National Center for Chronic and Noncommunicable Disease Control and Prevention, Chinese Center for Disease Control and Prevention, and the development process was based on insights from the literature review and the specific goals of this investigation. The questionnaire consists of three sections: (1) General information such as gender, age, ethnicity, region, educational level, occupation, marital status, place of residence, insurance coverage, smoking, alcohol consumption, comorbidities, duration of diabetes, and treatment methods. (2) Information about patients’ DKD-related tests in the past six months, including whether they have had fasting blood sugar tests, glycosylated hemoglobin tests, urine protein-to-creatinine ratio tests, and estimated glomerular filtration rate tests in the past year. (3) Patients’ awareness and assessment of DKD follow-up and screening, including their opinions on the importance of annual follow-ups to monitor kidney health, the ease of access to early screening services for diabetic kidney disease, the cost of diabetic kidney disease screening (approximately 50 RMB, before insurance reimbursement), and their willingness to undergo annual screening for diabetic kidney disease. The options for these questions are rated on a 1–5 scale, with five levels of evaluation. The supplementary file provides the content of the questionnaire.

### Sample size

In this study, the prevalence of diabetes in the Chinese population is π = 11%[6], the tolerance is α = 0.2, the statistical significance is α = 0.05, and the non-response rate is 15%. The required sample size is at least 952 people in each province. In order to facilitate on-site recruitment and organization and implementation, the sample size in each province is not less than 1,000 people.

### Data collection and statistical analysis

All investigators conducted one-on-one and face-to-face investigations with the respondents after two training sessions, and filled out the questionnaires by asking the respondents. The database was established with MS Excel, and the data were analysed with SAS 9.4 software.

When analysing the evaluation of DKD screening services, necessary for DKD follow-up was defined as the answers of “necessary”, “relatively necessary”, and “especially necessary”. DKD services easy to obtain was defined as the answers of “easy”, “relatively easy” and “extremely easy”. DKD service cheap was defined as the answers of “the price is acceptable”, “relatively cheap”, and “very cheap”. Acceptable DKD service was defined as the answers of “acceptable”, “more willing to accept”, and “especially willing to accept”. Southern regions included Hubei, Guangdong, and Chongqing. Northern regions included Tianjin, Heilongjiang, and Gansu.

### Ethics

This study was approved by the Ethics Review Committee of the National Center for Chronic and Noncommunicable Disease Control and Prevention Center, China CDC (conference review No. 202,112). All respondents signed informed consent.

## Results

### General characteristics of the survey object

The survey distributed a total of 6,713 questionnaires, with 6,230 valid questionnaires collected. The questionnaire’s validity rate is 92.81%. Among the surveyed population, 3,212 were female patients, accounting for 51.56% of the total, and 4,100 patients were aged 60 years and above, representing 65.81% of the respondents. Education levels varied, with 20.29% having education below the primary school level, 24.21% completing primary school, and 28.84% having education up to junior high school. In terms of the duration of diabetes, the proportions of patients with disease durations of less than 5 years, 5–9 years, and 10 years or more were 33.40%, 33.58%, and 33.02%, respectively (refer to Table 1). Additionally, the comorbidity rate among patients in the northern region stood at 57.48%, which was notably higher than the southern region at 46.58% (*P* < 0.01). Patients in the northern region who had been living with diabetes for over a decade accounted for 38.20%, which was also higher compared to the southern region at 28.03% (*P* < 0.01).

**Table 1:** General characteristics of the survey objects (*n* = 6230)

Characteristics	Number	Proportion (%)
Gender		
Male	3018	48.44
Female	3212	51.56
Age		
18–44 yrs	291	4.67
45-59yrs	1839	29.52
60 and above	4100	65.81
Ethics		
Han nationality	6179	99.18
Other nationality	51	0.82
Education level		
Under primary school	1264	20.29
Primary school	1508	24.21
Junior high school	1797	28.84
Senior high school	1205	19.34
College and above	456	7.32
Occupation		
Fisherman, agricultural, forestry, animal husbandry, fishery and production personnel	2132	34.22
Staff of enterprises and institutions	434	6.97
Retired	2082	33.42
Unemployed	918	14.74
Other	664	10.66
Marriage		
Unmarried	80	1.28
Married	5504	88.35
Divorced	91	1.46
Widowed	555	8.91
Residential		
Urban areas	3076	49.37
Rural areas	3154	50.63
^*^ Insurance		
Medical insurance for urban and rural residents	3898	62.57
Medical insurance for urban employees	2213	35.52
Medical assistance	36	0.58
At one’s own expense	471	7.56
Commercial insurance	52	0.83
Public medical care	14	0.22
Smoking		
Yes	946	15.18
No	5284	84.82
Alcohol drinking		
Yes	984	15.79
No	5246	84.21
Comorbidity		
Yes	3235	51.93
No	2995	48.07
^*^ Treatment		
Lifestyle treatment	4613	74.04
Oral medicine	5327	85.51
Insulin injection	2324	37.30
Traditional Chinese Medicine	517	8.30
Other	29	0.47
Duration of T2DM		
under five years	2081	33.40
5–9 years	2092	33.58
10 years and above	2057	33.02

Note^*^Multiple choices

### DKD-related testing practices

Within six months, 89.70% of the patients had fasting blood glucose measured, 21.12% of the patients had glycosylated haemoglobin measured, and only 13.11% and 9.34% of the patients had an ACR and eGFR measuredevery 12 months. Young people (18–44 years old), residents of northern areas, and patients with high education levels were tested for glycosylated hemoglobin within 6 months, ACR and eGFR test every 12 months with a higher frequency (all *P* < 0.05). Patients with long duration of diabetes had a higher frequency of measuring glycosylated haemoglobin within 6 months and eGFR every 12 months (all *P* < 0.05). However, middle-aged older adults (aged 45 years and above), rural residents, and patients with low education levels and comorbidities had higher frequency of testing fasting blood glucose within 6 months (all *P* < 0.05), see Table 2.

**Table 2:** DKD related testing practices [n(%)]

Characteristics	Fasting glucose testing within 6 months	HbA1C testing within 6 months	Annual ACR	Annual eGFR
Total	5588(89.70)	1316(21.12)	817(13.11)	582(9.34)
Gender				
Male	2690(89.13)	643(21.31)	413(13.68)	297(9.84)
Female	2898(90.22)	673(20.95)	404(12.58)	285(8.87)
*χ*2	2.01	0.12	1.67	1.72
*P*	0.16	0.73	0.20	0.19
Age				
18–44 yrs	234(80.41)	95(32.65)	52(17.87)	53(18.21)
45-59yrs	1641(89.23) ^a^	406(22.08) ^a^	257(13.97)	165(8.97) ^a^
60 and above	3713(90.56) ^a^	815(19.88) ^a^	508(12.39) ^a^	364(8.88) ^a^
*χ* ^2^	30.88	28.01	8.86	28.38
*P*	< 0.01	< 0.01	0.01	< 0.01
Residential				
Urban areas	2651(86.18)	679(22.07)	425(13.82)	301(9.79)
Rural areas	2937(93.12)	637(20.20)	392(12.43)	281(8.91)
*χ* ^2^	81.06	3.29	2.63	1.41
*P*	< 0.01	0.07	0.10	0.23
Location				
Southern	2864(90.20)	572(18.02)	205(6.46)	209(6.58)
Northern	2724(89.17)	744(24.35)	612(20.03)	373(12.21)
*χ* ^2^	1.82	37.53	251.84	58.20
*P*	0.18	< 0.01	< 0.01	< 0.01
Education level				
Under primary school	1175(92.96)	194(15.35)	116(9.18)	56(4.43)
Primary school	1344(89.12) ^b^	223(14.79)	146(9.68)	104(6.90)
Junior high school	1608(89.48) ^b^	402(22.37) ^b, c^	249(13.86) ^b, c^	191(10.63) ^b, c^
Senior high school	1078(89.46) ^b^	319(26.47) ^b, c^	194(16.10) ^b, c^	137(11.37) ^b, c^
College and above	383(83.99) ^b, c,d, e^	178(39.04) ^b, c,d, e^	112(24.56) ^b, c,d, e^	94(20.61) ^b, c,d, e^
*χ* ^2^	31.31	171.81	95.52	124.43
*P*	< 0.01	< 0.01	< 0.01	< 0.01
Comorbidity				
Yes	3026(93.54)	729(22.53)	444(13.72)	316(9.77)
No	2562(85.54)	587(19.60)	373(12.45)	266(8.88)
*χ* ^2^	107.6	8.04	2.20	1.44
*P*	< 0.01	< 0.01	0.14	0.23
Duration of T2DM				
Under five years	1855(89.14)	439(21.10)	269(12.93)	202(9.71)
5–9 years	1888(90.25)	359(17.16) ^f^	253(12.09)	161(7.70)
10 years and above	1845 (89.69)	518(25.18) ^f, g^	295(14.34)	219(10.65) ^g^
*χ* ^2^	1.39	40.06	4.69	11.15
*P*	0.50	< 0.01	0.10	< 0.01

Bonferroni’s correction for multiple comparisons was used. ^a^*P*<0.017 vs. 18–44 yrs. ^b^*P*<0.005 vs. Under primary school; ^c^*P*<0.005 vs. Primary school; ^d^*P*<0.005 vs. Junior high school; ^e^*P*<0.005 vs. Senior high school. ^f^*P*<0.017 vs. Under five years; ^g^*P*<0.017 vs. 5–9 years

### Evaluation of DKD screening services

Among all respondents, 84.43% of patients expressed a willingness to receive DKD services, while 79.13% and 78.11% believed that DKD follow-up was essential and that the cost of DKD services was affordable. However, only 60.18% considered DKD services easy to access. When examining various demographic characteristics, male patients with higher education levels demonstrated a stronger conviction that DKD follow-up was necessary, that DKD services were reasonably priced, and that they were more amenable to accepting DKD screening services (*P* < 0.01). Comparatively, patients with comorbidities, in contrast to those without comorbid conditions, expressed a stronger belief in the necessity of DKD follow-up, perceived DKD services as more cost-effective, and displayed a higher willingness to accept DKD screening services (*P* < 0.01). Moreover, urban patients, as opposed to their rural counterparts, held the opinion that the availability of DKD services was superior, considered DKD services more affordable, and exhibited a greater readiness to embrace DKD screening services (*P* < 0.05). See Table 3.

**Table 3:** Assessment of DKD services [n(%)]

Characteristics	DKD follow up necessity	DKD services availability	DKD services price cheap	DKD services acceptability
Total	4930(79.13)	3749(60.18)	4866(78.11)	5260(84.43)
Gender				
Male	2436(80.72)	1862(61.70)	2433(80.62)	2603(86.25)
Female	2494(77.65)	1887(58.75)	2433(75.75)	2657(82.72)
*χ*2	8.88	5.64	21.57	14.73
*P*	< 0.01	0.02	< 0.01	< 0.01
Age				
18–44 yrs	235(80.76)	189(64.95)	245(84.19)	251(86.25)
45-59yrs	1491(81.08)	1123(61.07)	1472(80.04)	1578(85.81)
60 and above	3204(78.15) ^a^	2437(59.44)	3149(76.80) ^a, b^	3431(83.68)
*χ* ^2^	7.09	4.30	14.40	5.13
*P*	0.03	0.12	< 0.01	0.08
Residential				
Urban areas	2434(79.13)	1890(61.44)	2528(82.18)	2658(86.41)
Rural areas	2496(79.14)	1859(58.94)	2338(74.13)	2602(82.50)
*χ* ^2^	< 0.01	4.07	59.11	18.13
*P*	0.99	0.04	< 0.01	< 0.01
Location				
Southern	2491(78.46)	1764(55.56)	2434(76.66)	2686(84.60)
Northern	2439(79.84)	1985(64.98)	2432(79.61)	2574(84.26)
*χ* ^2^	1.79	57.61	7.90	0.14
*P*	0.18	< 0.01	< 0.01	0.71
Education level				
Under primary school	932(73.73)	648(51.27)	892(70.57)	1008(79.75)
Primary school	1150(76.26)	841(55.77)	1090(72.28)	1192(79.05)
Junior high school	1451(80.75) ^c, d^	1120(62.33) ^c, d^	1456(81.02) ^c, d^	1564(87.03) ^c, d^
Senior high school	1004(83.32) ^c, d^	807(66.97) ^c, d^	1023(84.90) ^c, d^	1075(89.21) ^c, d^
College and above	393(86.18) ^c, d^	333(73.03) ^c, d,e^	405(88.82) ^c, d,e^	421(92.32) ^c, d,e^
*χ* ^2^	59.20	112.20	143.92	106.20
*P*	< 0.01	< 0.01	< 0.01	< 0.01
Comorbidity				
Yes	2632(81.36)	1941(60.00)	2580(79.75)	2803(86.65)
No	2298(76.73)	1808(60.37)	2286(76.33)	2457(82.04)
*χ* ^2^	20.21	0.09	10.67	25.13
*P*	< 0.01	0.77	< 0.01	< 0.01
Duration of T2DM				
Under five years	1595(76.65)	1195(57.42)	1600(76.89)	1733(83.28)
5–9 years	1672(79.92) ^f^	1271(60.76)	1644(78.59)	1765(84.37)
10 years and above	1663(80.85) ^f^	1283(62.37) ^f^	1622(78.85)	1762(85.66)
*χ* ^2^	12.24	11.01	2.76	4.47
*P*	< 0.01	< 0.01	0.25	0.11

Bonferroni’s correction for multiple comparisons was used. ^a^*P*<0.017 vs. 18–44 yrs; ^b^*P*<0.017 vs. 45–59 yrs. ^c^*P*<0.005 vs. Under primary school; ^d^*P*<0.005 vs. Primary school; ^e^*P*<0.005 vs. Junior high school. ^f^*P*<0.017 vs. Under five years

### Factors related to the practice of DKD detection

Logistic regression analysis showed that middle-aged and elderly (45 years old and above, patients residing in rural areas) were more likely to take fasting blood glucose testing every 6 months. For glycosylated hemoglobin testing every 6 months, being female was a favorable factor for testing. In contrast, geographic location, education level below junior college, no comorbidities, and duration of diabetes within 10 years were risk factors for testing. For the ACR and eGRF tests, southern geographical location and the education level below the college were the risk factors for testing, see Table 4.

**Table 4:** DKD screening services related factors

Characteristics	Fasting glucose testing within 6 months		HbA1C testing within 6 months		Annual ACR		Annual eGFR	
	OR(95%CI)	P	OR(95%CI)	P	OR(95%CI)	P	OR(95%CI)	P
Gender								
Male	Ref		Ref		Ref		Ref	
Female	1.10(0.93 ~ 1.30)	0.28	1.16(1.02 ~ 1.32)	0.03	0.97(0.83 ~ 1.14)	0.73	1.07(0.90 ~ 1.28)	0.44
Age								
18–44 yrs	Ref		Ref		Ref		Ref	
45-59yrs	1.73(1.22 ~ 2.46)	< 0.01	0.78(0.58 ~ 1.04)	0.09	1.14(0.80 ~ 1.63)	0.46	0.65(0.45 ~ 0.94)	0.02
60 and above	1.94(1.36 ~ 2.76)	< 0.01	0.76(0.57 ~ 1.02)	0.06	1.23(0.86 ~ 1.76)	0.25	0.81(0.56 ~ 1.17)	0.26
Residential								
Urban areas	Ref		Ref		Ref		Ref	
Rural areas	2.36(1.96 ~ 2.83)	< 0.01	1.12(0.98 ~ 1.27)	0.11	1.02(0.87 ~ 1.20)	0.79	1.16(0.97 ~ 1.40)	0.11
Location								
Northern	Ref		Ref		Ref		Ref	
Southern	1.19(1.00 ~ 1.42)	0.05	0.86(0.76 ~ 0.98)	0.02	0.30(0.25 ~ 0.35)	< 0.01	0.60(0.50 ~ 0.73)	< 0.01
Education level								
College and above	Ref		Ref		Ref		Ref	
Under primary school	1.32(0.91 ~ 1.91)	0.15	0.29(0.22 ~ 0.38)	< 0.01	0.38(0.28 ~ 0.53)	< 0.01	0.19(0.13 ~ 0.29)	< 0.01
Primary school	0.99(0.71 ~ 1.39)	0.96	0.30(0.23 ~ 0.39)	< 0.01	0.48(0.36 ~ 0.65)	< 0.01	0.35(0.25 ~ 0.49)	< 0.01
Junior high school	1.24(0.91 ~ 1.71)	0.18	0.49(0.39 ~ 0.62)	< 0.01	0.60(0.45 ~ 0.78)	< 0.01	0.54(0.40 ~ 0.72)	< 0.01
Senior high school	1.30(0.94 ~ 1.81)	0.12	0.59(0.46 ~ 0.75)	< 0.01	0.63(0.47 ~ 0.82)	< 0.01	0.54(0.40 ~ 0.73)	< 0.01
Comorbidity								
Yes	Ref		Ref		Ref		Ref	
No	0.41(0.34 ~ 0.49)	< 0.01	0.85(0.75 ~ 0.96)	0.01	1.00(0.86 ~ 1.17)	0.97	0.92(0.77 ~ 1.10)	0.38
Duration of T2DM								
10 years and above	Ref		Ref		Ref		Ref	
under five years	1.00(0.81 ~ 1.24)	0.98	0.81(0.69 ~ 0.94)	< 0.01	1.05(0.86 ~ 1.26)	0.65	0.97(0.78 ~ 1.20)	0.75
5–9 years	1.04(0.84 ~ 1.29)	0.71	0.67(0.57 ~ 0.78)	< 0.01	1.00(0.82 ~ 1.20)	0.96	0.81(0.65 ~ 1.00)	0.05

*Note* OR < 1 is risk factor, OR > 1is protect factor

## Discussion

This study showed that the fasting blood glucose detection rate of the surveyed subjects was high, reaching 89.70%, but the glycosylated hemoglobin (HbA1c), ACR, and eGFR testing was low at only 21.12%, 13.11%, and 9.34%, respectively. Fasting blood glucose testing is the only management service testing item for diabetic patients that is clearly stipulated and has assessment indicators in the national basic public health service standards in China [10]. The basic public health primary service requires fasting blood glucose measurement every 12 months for people at high risk of diabetes, free fasting blood glucose measurement and face-to-face follow-up every 3 months for patients diagnosed with T2DM. In 2009, the State Council of China issued the “Opinions on Deepening the Reform of the Medical and Health System”, and China government officially launched the implementation of the basic public health service project, focusing on patients with chronic diseases as a key group, and providing the most basic public health services free of charge. The per capita basic public health service expenditure increased from 15 yuan RMB in 2010 to 79 yuan RMB in 2021. Therefore, it is speculated that the sufficient investment of policies and funds is one of the guarantee factors for the high fasting blood glucose detection rate of Chinese patients with T2DM. Research indicates that over the past few decades, China has made progress in improving health literacy. The level of health literacy has increased from 6.48% in 2008 to 23.15% in 2020 [12]. The improvement in health literacy has also positively impacted screening efforts.

HbA1c testing is essential to ensure the management effect of diabetic patients. HbA1c testing every 3–6 months is one of the important monitoring measures in the management of diabetic patients [13]. The results of this survey suggest that the detection rate of HbA1c in Chinese patients with T2DM is very low. Results of a study conducted in the United States showed that 66% of patients were unaware of their most recent HbA1c test results, and only 25% were able to accurately report the value. Reports from the U.S. Center for Disease Control and Prevention indicate that less than 30% of patients were aware of HbA1c testing, and even fewer were aware of HbA1c control targets [14, 15]. A study by Heisler et al. showed that 66% of patients were unaware of their most recent HbA1c results, and only 25% reported accurate HbA1c results [16]. The above results suggest that there is much room for improvement in HbA1c cognition and detection practice in patients with T2DM.

The results of this study showed that the annual proportion of ACR and eGFR testing in Chinese patients with T2DM was very low. A large gap exists between this status and the guideline that “patients with type 2 diabetes should receive nephropathy-related examinations every year" [9]. Brazil, Germany, and the United States have also reported low adherence to kidney disease-related testing [17]. The existence of multiple factors influences adherence to clinically relevant practice guidelines for disease management, such as patient awareness of health care, physician familiarity with medical conditions and guideline recommendations, physician awareness of following guidelines, patient values, physician or patient perceptions of care, and preference for intervention services, etc. [18–20]. The results of this study show that the proportion of HbA1c, ACR and eGFR testing in young, northern, highly educated, and long-term type 2 diabetic patients in China was relatively high. The prevalence of diabetes in China is higher in northern regions than in southern regions [21]. Therefore, compared with the south, the provision of diabetes education and management services in the north may be relatively sufficient, which may be the reason for the relatively high proportion of DKD-related testing in patients in the north. In addition, this survey also suggests that the subjects surveyed in northern have a relatively long disease course and relatively common comorbidities, which are also be the reason for the findings. As the disease course progresses, patients are more likely to receive DKD-related examinations and management services, which may also be a factor in higher detection rates in patients with a long disease course. The high proportion of DKD-related testing in young and highly educated patients may be related to the higher level of health literacy among young and highly educated populations in China [22].

Evidence from a systematic review suggests that screening for CKD by eGFR and/or proteinuria in high-risk groups (patients with diabetes or hypertension) is cost-effective (<$50,000/QALY) [23]. However, through a survey of Chinese patients with T2DM, it was found that the respondents generally believed that DKD prevention and screening services were not easy to obtain. Compared with urban patients, rural patients believed that it was more difficult to obtain DKD screening services. Although the urban and rural coverage of medical care in China has reached more than 95%[24], there are more challenges and difficulties in providing medical care services in rural areas than in cities, such as income and economic factors, lack of medical care services in rural areas, as well as traditional values ​​of rural residents (e.g., reluctance to seek medical services unless severely compromised by health problems, etc.), high out-of-pocket costs, and complicated reimbursement procedures, etc. [25–27]. Based on this, possible solutions in rural areas are to reduce the financial barriers to the reimbursement of preventive services, increase the supply of primary health care services and personnel, and raise awareness of the importance of preventive care services.

### Limitation

This study used a cross-sectional survey and so there may be recall bias of the respondents. To make up for this limitation, the researchers selected contracted family physicians who were familiar with the subjects’ lifestyle and disease history as field investigators. During the investigation, in order to facilitate the respondents to understand professional terms, the investigators explained the content of DKD-related tests in plain language. At the same time, investigators compared the survey results (such as diabetes duration, comorbidities, and medical tests) with subsequent registration data in the basic public health system to confirm the true situation of the survey subjects.

## Conclusion

The results of this survey demonstrated that the proportion tested for urine protein to creatinine ratio, estimated glomerular filtration rate, and glycosylated hemoglobin in Chinese patients with T2DM was very low, and the proportion of fasting blood glucose testing was high. Patients with T2DM in rural areas, in southern areas, with low education level, and with disease of short duration have lower detection rates for DKD prevention and treatment. Based on the results of this study, the following measures are recommended: strengthen the population’s awareness of the importance of DKD preventive care services, increase the supply of DKD services in primary health care institutions, and consider the detection of patients with T2DM with different population characteristics when allocating relevant resources.

### Electronic supplementary material

Below is the link to the electronic supplementary material.

Supplementary Material 1

## Data Availability

The datasets generated and analysed during the current study are not publicly available as the informed consent applied only for the use by the research team. If desired, the data can be viewed and reviewed together with the corresponding author.
