# Supplementary material for: Diabetic kidney disease screening status and related factors: a cross-sectional study of patients with type 2 diabetes in six provinces in China
Source: BMC Health Serv Res. 2024 Apr 19;24:489. doi: 10.1186/s12913-024-10938-9 (PMC11031931; doi:10.1186/s12913-024-10938-9)
Supplement: Supplementary file 1 — Supplementary Material 1 [file 12913_2024_10938_MOESM1_ESM.pdf]

【Diabetes Patient Survey】

1. Home Address: __________ [Fill in the blank] *

2. Date of Birth: ___ [Fill in the blank] *

3. Gender: [Single-choice question] *
   ○ Male
   ○ Female

4. Ethnicity: [Single-choice question] *

○ Han
○ Zang
○ Hui
○ Tu
○ Sala
○ Other (Specify) _________________ *

5. Educational Level: [Single-choice question] *

○ No formal education
○ Did not complete primary school
○ Completed primary school
○ Completed junior high school
○ Completed high school, vocational school, technical school
○ Completed College
○ Completed undergraduate studies
○ Postgraduate and above

6. Occupation: [Single-choice question] *

○ Agricultural, forestry, animal husbandry, and fishery production personnel
○ Production and transportation equipment operators and related personnel
○ Business and service industry personnel
○ Leaders of state organs, and mass organizations, enterprises, and institutions
○ Office workers and related personnel
○ Professional and technical personnel
○ Military personnel
○ Other laborers
○ Students
○ Unemployed
○ Housework
○ Retired

7. Your current marital status: [Single-choice question] *

○ Unmarried

○ Married
○ Divorced
○ Widowed

8. In the past year, what type of medical insurance have you participated in? [Multiple-choice question] *

□ Rural Cooperative Medical Care
□ Urban Employee Medical Insurance
□ Urban Resident Medical Insurance
□ Medical Assistance*
□ Self-pay
□ Commercial Insurance
□ Public Medical Care

9. Do you currently smoke? [Single-choice question] *

○ Do not smoke (Skip to question 61)
○ Yes, but not every day
○ Yes, every day

10. In the past year, have you consumed alcohol? [Single-choice question] *

○ Have not consumed
○ Have consumed

11. In which year were you diagnosed with diabetes? __________ [Fill in the blank] *

12. Have you been informed by doctors from secondary or higher-level hospitals that you have the following chronic diseases? [Matrix single-choice question] *

Yes No
○ Chronic Kidney Disease
○ Gout
○ Rheumatic Immunological Diseases
○ Polycystic Kidney Disease
○ Coronary Heart Disease
○ Cerebral Infarction (Stroke)
○ Myocardial Infarction
○ Hypertension
○ Cancer
○ Chronic Obstructive Pulmonary Disease (e.g., Chronic Bronchitis, Emphysema)

13. How do you treat diabetes? (Choose single or multiple options) [Multiple-choice question] *

○ Lifestyle adjustments
○ Oral Western medicine
○ Insulin injection
○ Oral traditional Chinese medicine

○ Other __________________*

14. Have you measured fasting blood sugar in the last six months? [Single-choice question] *
○ Yes
○ No

15. Have you measured glycosylated hemoglobin in the last six months? [Single-choice question] *
○ Yes
○ No

16. Have you undergone Urine Albumin-to-Creatinine Ratio (UACR) testing? [Single-choice question] *
○ Yes, at least once a year
○ Yes, but not every year
○ Never done
○ Not sure

17. Have you undergone estimated Glomerular Filtration Rate (eGFR) testing? [Single-choice question] *
○ Yes, at least once a year
○ Yes, but not every year
○ Never done
○ Not sure

18. Do you think it is necessary to have an annual follow-up to understand your kidney health? [Single-choice question] *
○ Absolutely unnecessary
○ Not very necessary
○ Necessary
○ Quite necessary
○ Absolutely necessary

19. Do you find it easy to access early screening services for diabetic kidney disease? [Single-choice question] *
○ Not very easy
○ Somewhat easy
○ Easy
○ Quite easy
○ Very easy

20. Do you think the cost of diabetes kidney disease screening is expensive (about 50 yuan, before medical insurance reimbursement)? [Single-choice question] *
○ Too expensive

○ Quite expensive
○ Affordable
○ Quite cheap
○ Very cheap

21. Would you be willing to undergo diabetes kidney disease screening once a year? [Single-choice question] *

○ Absolutely unwilling
○ Not very willing
○ Willing
○ Quite willing
○ Very willing
